# Interplay between SFTSV and host innate immunity: Viral evasion mechanisms, pathogenesis, and therapeutic perspectives

**DOI:** 10.1016/j.isci.2026.117588

**Published:** 2026-09-24

**Authors:** Xue Ma, Sai Yang, Zi-Ming Wu, Bo Pang, Longfei Kang, Lingxue Shi, Xia Jiang, Chuan-min Zhou

**Affiliations:** 1 Hebei Key Laboratory of Colorectal Cancer Precision Diagnosis and Treatment, The First Hospital of Hebei Medical University, Shijiazhuang, China; 2 Key Laboratory of Maternal and Fetal Medicine of Hebei Province, Prenatal Diagnosis Center, Shijiazhuang Obstetrics and Gynecology Hospital, Shijiazhuang, China; 3 Department of Nursing, Union Hospital, Tongji Medical College, Huazhong University of Science and Technology, Wuhan, Hubei 430022, China; 4 State Key Laboratory of Virology, School of Public Health, Wuhan University, Wuhan, China

**Keywords:** bunyavirus, SFTSV, innate immunity, immune escape, pattern recognition receptor, programmed cell death

## Abstract

Severe fever with thrombocytopenia syndrome (SFTS), caused by SFTSV, is an emerging tick-borne disease with substantial mortality in East Asia and increasing geographic reach. Here, we provide an updated conceptual framework that integrates classic host recognition via DNA and RNA sensors accompanied with the multifaceted immune evasion strategies orchestrated by the viral components. A central focus is that SFTSV utilizes host receptors for attachment and internalization while hijacking autophagy-related membranes to promote assembly and egress. Its NSs protein forms autophagic inclusion bodies that sequester and degrade innate immune factors, whereas NP and Gn interfere with RIG-I-MAVS and cGAS-STING signaling through autophagy-associated mechanisms. We also examine NS-driven inflammatory regulation and evidence that the microbiota shapes virus transmission and antiviral responses. Collectively, unraveling these intricate host-virus interactions will be pivotal for advancing targeted therapeutic strategies against SFTS.

## Graphical abstract

**Figure:**
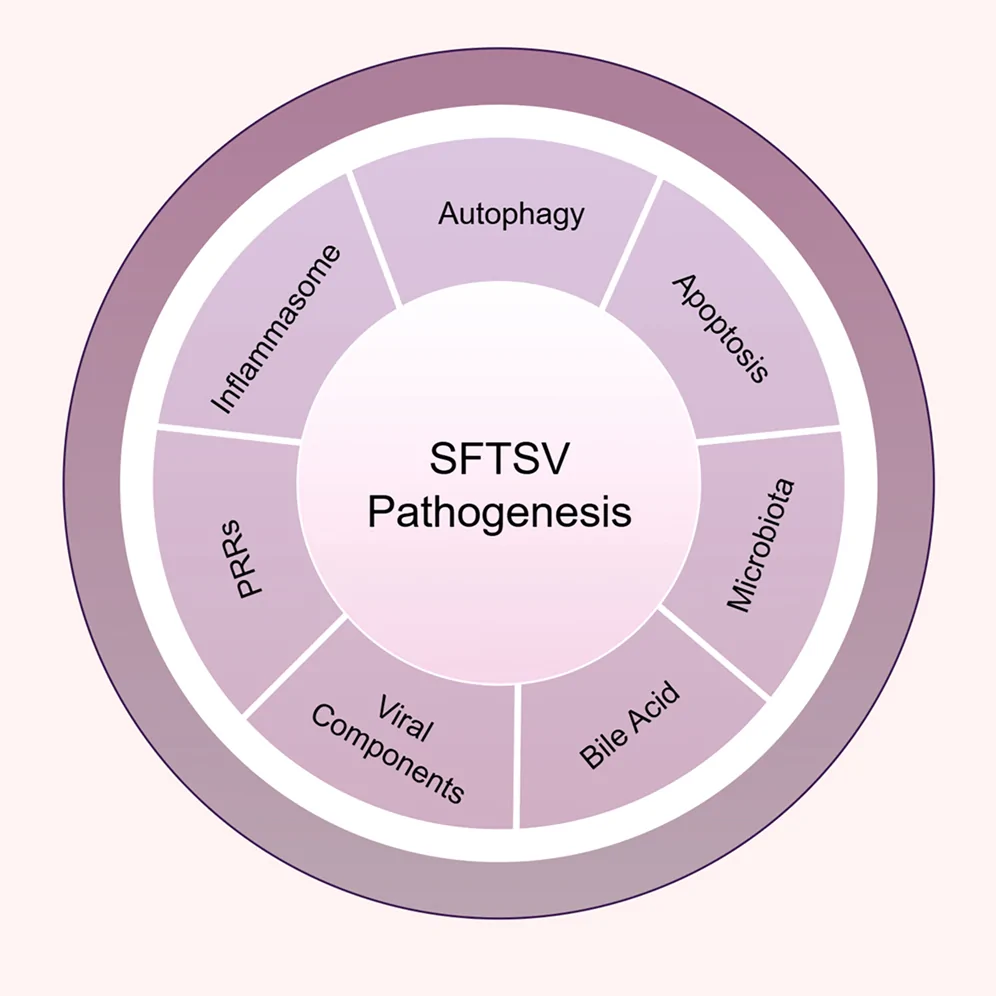

## Teaser

Biological sciences; Immunology; Microbiology; Virology

## Introduction

Severe fever with thrombocytopenia syndrome virus (SFTSV), also referred to as *Dabie bandavirus*, is a highly pathogenic tick-borne virus classified within the genus *Bandavirus*, family *Phenuiviridae*, and order *Bunyavirale*. Initially identified in rural regions of Hubei and Henan provinces in Central China in 2009, the SFTSV epidemics have progressively spread geographically.^1^ SFTSV infection has been documented in 27 provinces across China, as well as in South Korea, Japan, Thailand, Vietnam, and Pakistan, raising global public health concerns.^2^^,^^3^^,^^4^^,^^5^^,^^6^^,^^7^^,^^8^ The virus causes a severe viral hemorrhagic fever known as SFTS, characterized by prominent clinical manifestations such as fever, thrombocytopenia, leukocytopenia, hemorrhage, gastrointestinal symptoms, and even multiple organ failure. The average case fatality rate ranges from 16% to 20%, reaching as high as 45.7% in some cohorts.^1^^,^^9^ Of note, thrombocytopenia has been recognized as a critical risk factor for mortality.^10^ Recent research has elucidated that SFTSV interacts with circulating platelets via glycoprotein VI, resulting in platelet activation. This activation predisposes platelets to aggregate and adhere to endothelial cells, thereby facilitating platelet clearance through enhanced platelet-macrophage interactions, which ultimately leads to thrombocytopenia.^11^^,^^12^ Furthermore, as a polytropic virus, SFTSV is capable of infecting a diverse array of human immune cells, including monocytes, T cells, B cells, and natural killer (NK) cells.^13^

SFTSV is predominantly transmitted via bites from infected ticks, with *Haemaphysalis longicornis* identified as the main tick reservoir and vector.^1^^,^^14^ Wild animals (e.g., mice, hedgehogs, and migratory birds), livestock (e.g., goats, cattle), and companion animals (e.g., cats, dogs) act as potential amplifying hosts.^15^ Importantly, migratory birds may play a role in the transoceanic spread of SFTSV.^16^ Although tick-to-human transmission constitutes the primary mode of transmission, instances of animal-to-human and human-to-human transmission have been documented.^15^ Human-to-human transmission may occur through exposure to blood, body fluids, or aerosols, posing a significant risk to caregivers and healthcare workers.^17^^,^^18^^,^^19^^,^^20^^,^^21^ Importantly, a recent study suggests that while bats exhibit seropositivity but remain RNA negative for SFTSV, viral RNA segments were detected in ticks collected near bat habitats. This points to potential ecological interactions involving bats, ticks, and other wildlife species.^22^

Despite recent advancements, a comprehensive understanding of the arms race between SFTSV and the host innate immunity remains incomplete. The aim of this review is to delineate the molecular mechanisms underlying both innate immune recognition and the multifaceted evasion strategies orchestrated by SFTSV or viral components. Specifically, we highlight recent breakthroughs in autophagy-mediated viral assembly and egress, inflammatory responses modulation, and gut homeostasis interaction (Figure 1; Table 1). Ultimately, by synthesizing these complex host-pathogen interactions, we seek to provide an updated conceptual framework that not only identifies current knowledge gaps but also accelerates the development of novel host-directed therapeutics against SFTSV infection.

**Figure 1:**
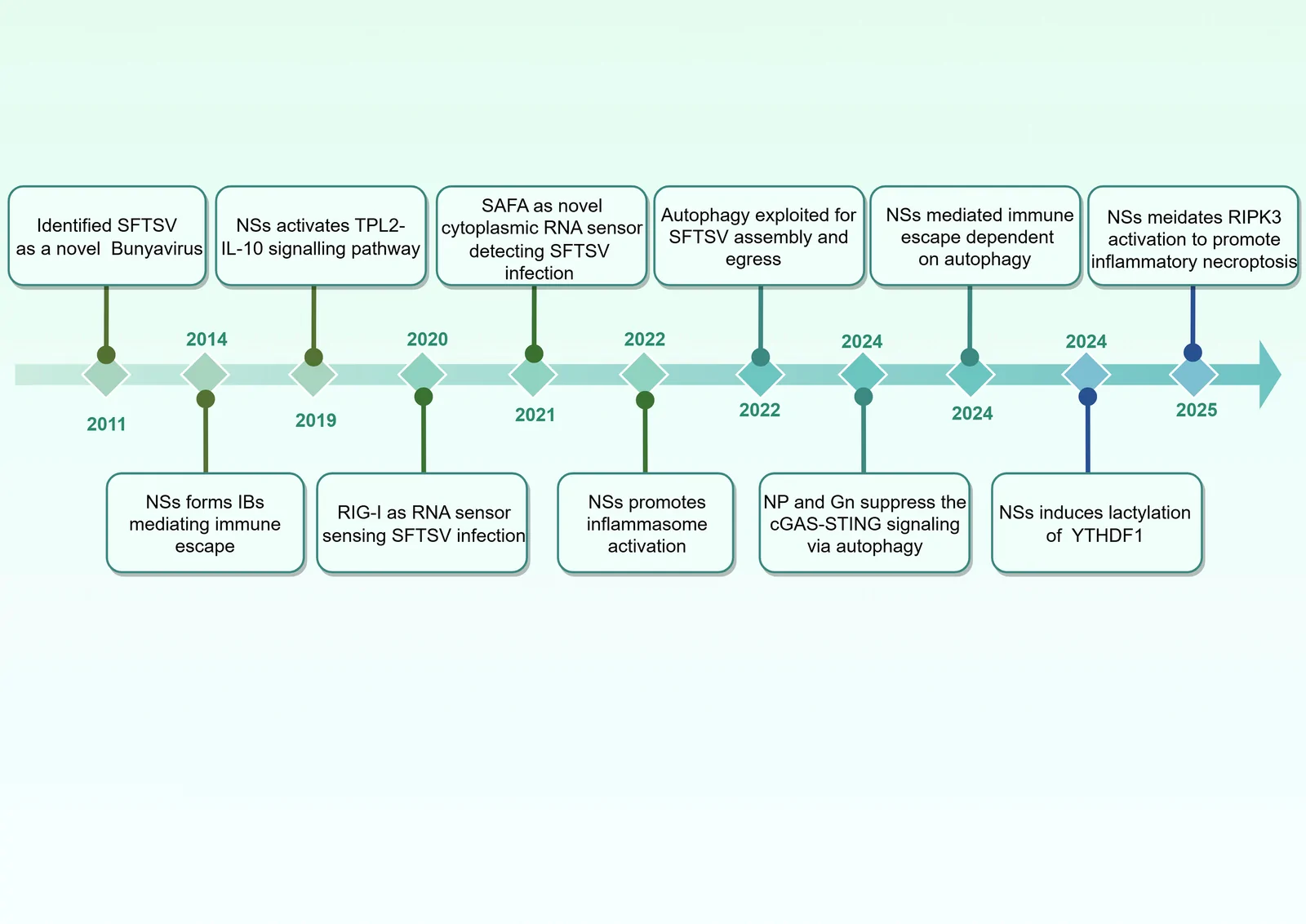
Timeline of important research in SFTSV and innate immunity

**Table 1:** Summary of host innate immune factors targeted by SFTSV NSs

Host factor	Pathway affected	Mechanism of antagonism
RIG-I, TRIM25	RNA sensing	Sequestration into IBs^23^^,^^24^
SAFA	RNA sensing	Autophagic degradation^25^
TBK1, IKKε, IRF3, IRF7	Type I IFN induction	Sequestration into IBs^26^^,^^27^^,^^28^
STAT1, STAT2	JAK-STAT cascade	Sequestration into IBs^29^^,^^30^
mTOR, Beclin1, Vimentin	Autophagy	Promoting autophagy initiation complex^31^^,^^32^^,^^33^
RIPK3	Necroptosis/apoptosis	Facilitates RIPK3 autophosphorylation^34^
NLRP3, DPP8/9	Inflammasome	Promoting inflammasome assembly^35^^,^^36^
ABIN2	IL-10 induction	Promoting the assembly of the TPL2/ABIN2/p105 complex^37^
LSm14A	RNA processing	Sequestration into IBs^38^
CDK1	Cell cycle	Sequestration into IBs^39^
AGO2	RNAi pathway	Sequestration into IBs^40^

## SFTSV components and life cycle

SFTSV is an enveloped, icosahedral spherical RNA virus with a virion diameter of 80–100 nm. The virus comprises three distinct components: the large (L), medium (M), and small (S) segments. Each RNA segment forms panhandle-like secondary structures through terminal complementary sequences.^1^ The L segment encodes an RNA-dependent RNA polymerase (RdRp), responsible for viral genome transcription and replication. The M segment encodes a glycoprotein precursor that undergoes proteolytic cleavage by host enzymes, generating two glycoproteins (Gn and Gc). These glycoproteins mediate virion attachment to host cell receptors and subsequent membrane fusion. The S segment employs an ambisense coding strategy. Its positive-sense strand translates into nonstructural proteins (NSs), which are critical virulence factors involved in immune evasion, while the complementary strand encodes the nucleoprotein (NP). The NP interacts with viral RNA and RdRp to form ribonucleoprotein (RNP) complexes, which typically serve as functional units for viral replication and assembly. Remarkably, recent studies have demonstrated that Gn and NP possess the ability to subvert the innate immune response, akin to the function of NSs.

The life cycle of SFTSV can be delineated into 5 main phases: attachment, internalization, replication, assembly, and exocytosis.^41^ SFTSV employs a collaborative multi-receptor strategy for host cell entry. The C-type lectin DC-SIGN functions as a receptor for SFTSV Gn/Gc-driven entry into dendritic cells,^42^^,^^43^ while NMMHC-IIA, C-C motif chemokine receptor 2 (CCR2), and pro-low-density lipoprotein receptor-related protein 1 (LRP1) coordinate viral attachment and internalization through Gn-specific interactions.^44^^,^^45^^,^^46^ Notably, a phosphatidylinositol 3-kinase (PI3K)-phospholipase C (PLC)-dependent micropinocytosis is utilized via interaction with the entry receptor AXL.^47^ Upon binding of SFTSV glycoproteins to cell receptors, the virus is transported via clathrin-mediated endocytosis into Rab5a^+^ early endosomes.^41^^,^^48^ Early endosomes undergo acidification and transition into Rab7a^+^, LAMP-1^+^ late endosomes that facilitate viral uncoating.^48^ Of note, sorting nexin 11 (SNX11) is involved in the intracellular endosomal trafficking and penetrating process of SFTSV infection.^49^ The acidic conditions within late endosomes mediate a conformational change of glycoproteins, promoting viral envelope-endosome membrane fusion and resulting in cytoplasmic release of RNPs. These released RNPs initiate primary transcription processes before being trafficked to the rough endoplasmic reticulum for the coordinated execution of viral replication, transcription, and protein synthesis.^50^^,^^51^ Additionally, RNAs related to the WNT-CTNNB1 signaling pathway could be utilized as a primer “pool” for SFTSV cap-snatching.^52^^,^^53^ To generate progeny virions, bunyaviruses typically exploit the Golgi apparatus for assembly and employ Golgi-derived secretory vesicles for egress. Recent evidence indicates that SFTSV hijacks autophagy-related membrane trafficking pathways for both assembly and egress.^54^ The ERGIC and Golgi apparatus, rather than the ER, serve as the membrane sources for autophagosome formation and function as platforms for SFTSV assembly. Additionally, the fusion of autophagy-dependent vesicles with lysosomes is pivotal for SFTSV exocytosis. Parallel studies reveal a complementary dissemination strategy whereby CD63^+^ extracellular vesicles facilitate the egress of SFTSV.^55^

## Host recognition of SFTSV infection

The innate immune response serves as the primary defense mechanism against viral infections, primarily through the activation of nucleic acid pattern recognition receptors and subsequent activation of type I interferon (IFN) signaling pathways. Proper activation of IFN signaling pathways at low to moderate viremia levels contributes to survival during SFTSV infection. In contrast, excessive activation of these pathways at high viremia levels leads to the aberrant induction of multiple inflammatory cytokines and chemokines, resulting in lethal disease outcomes.^13^^,^^56^^,^^57^ Within the context of SFTSV infection, the RIG-I-MAVS signaling pathway is capable of initiating type I IFN signaling by detecting 5′-triphosphate genomic RNAs and packaged RNPs.^23^^,^^57^^,^^58^ Conversely, MDA5 exhibits a limited capacity for SFTSV recognition, with the underlying mechanism remaining elusive.^23^ Furthermore, SFTSV infection activates TLR7/8-MyD88 signaling, with TLR7 specifically influencing viral replication.^59^ Beyond conventional RNA sensors, the nuclear dsRNA-binding protein SAFA relocates to the cytoplasm through interaction with SFTSV NP, thereby activating STING-mediated type I IFN signaling by detecting viral RNA.^60^ Notably, the DNA sensor cGAS enhances antiviral defense by detecting relocated mitochondrial DNA (mtDNA), thereby initiating type I IFN signaling.^61^^,^^62^ Although *cGAS*^−/−^ mice exhibit increased susceptibility to SFTSV infection, SFTSV infection is not lethal in these mice.^61^

## SFTSV viral components induce autophagy for immune escape

To evade immune detection, SFTSV has evolved mechanisms to circumvent antiviral defenses by disrupting the type I IFN signaling, with the SFTSV NSs being the most extensively studied. The SFTSV NSs is capable of forming inclusion bodies (IBs), also referred to as viroplasm-like structures (VLSs), which facilitate immune evasion by sequestering downstream signaling molecules within the IBs (Figure 2A). The presence of the NSs PXXP motif is essential for the formation of IBs.^63^ Studies have identified various IFN-related molecules, such as TRIM25, RIG-I, SAFA, TBK1, IKKε, IRF3, IRF7, STAT1, and STAT2, as well as LSm14A involved in RNA processing, CDK1 involved in cell cycle regulation, AGO2 involved in the RNAi pathway, being sequestered into IBs, thereby attenuating the innate immune response.^23^^,^^24^^,^^26^^,^^27^^,^^28^^,^^29^^,^^30^^,^^38^^,^^39^^,^^40^^,^^63^^,^^64^^,^^65^^,^^66^ In addition, NSs interacts with the RIPK3 kinase domain to form the NSs-RIPK3 complex, which facilitates RIPK3 autophosphorylation and subsequent necroptosis activation in an RHIM-independent manner^34^(Figure 2B). Concurrently, the NSs-RIPK3 complex inhibits RIPK3-mediated apoptosis.^34^^,^^59^ IBs are essential for the activation of necroptosis and the inhibition of apoptosis, while these processes are critical for viral replication and pathogenesis^34^(Figure 1).

**Figure 2:**
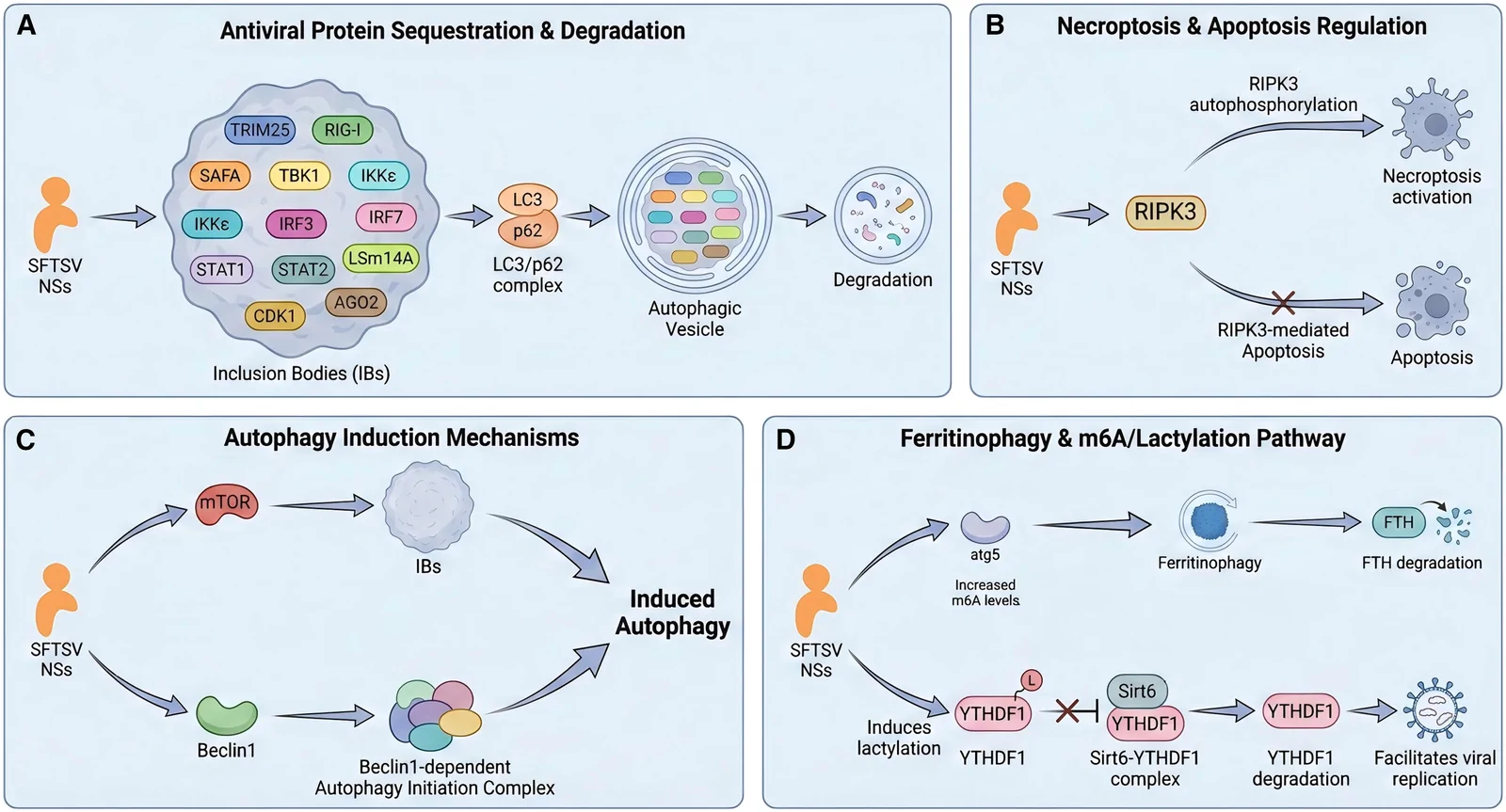
Multiple immune evasion strategies of SFTSV NSs SFTSV NSs facilitate immune escape by triggering the formation of IBs and inducing various programmed cell death or degradation pathways, including autophagy, ferritinophagy, and necroptosis.

It has been mentioned that autophagy vesicles provide a platform for both SFTSV assembly and egress.^54^ In addition, a novel model of immune evasion has been proposed, wherein SFTSV and its NSs induce complete autophagy and sequester host antiviral proteins within autophagic vesicles for degradation, thereby evading host innate immune responses^31^(Figure 2C). SFTSV NSs has been demonstrated to sequester mTOR into IBs, attenuate the inhibitory effect of vimentin on Beclin1, and promote the formation of a Beclin1-dependent autophagy initiation complex through interaction with Beclin1, thereby inducing autophagy.^31^^,^^32^^,^^33^ In addition, SFTSV infection and SFTSV NSs promotes the stability and oligomerization of p62, a phenomenon induced by the interaction of SFTSV NSs with TRIM21.^66^ In view of immune escape, SFTSV NSs facilitates the sequestration of antiviral proteins, such as TRIM25, RIG-I, NF-κB, SAFA, and DPP8/9, into autophagic vesicles via interaction with LC3 and p62, leading to their degradation and evasion of antiviral immune responses.^25^^,^^31^^,^^35^^,^^65^ The 8A mutant (S_97_xLRWPxG_104_) of NSs has been shown to reduce the induction of Beclin1-dependent autophagy flux and further degradation of antiviral immune proteins.^31^ Although the loss of the IBs motif does not completely abolish autophagy induction, the IBs motif plays a significant role in this process.^31^ Also, the tryptophan-to-tyrosine mutation at position 61 of NSs perturbs autophagy flux.^67^ Interestingly, recent research shows that NSs triggers ferritinophagy and induces FTH degradation by increasing the m^6^A levels of *atg5*,^68^ and NSs induces the lactylation of the m^6^A reader protein YTHDF1, thereby inhibiting the formation of the Sirt6-YTHDF1 complex, which leads to the degradation of YTHDF1 and consequently facilitates viral replication^69^^,^^70^(Figure 2D).

In addition to NSs, other components of the SFTSV, specifically the NP and Gn, play a critical role in inducing autophagy and facilitating immune evasion^54^^,^^61^^,^^62^^,^^71^(Figure 3). The SFTSV NP initiates mitophagy to degrade MAVS, thereby inhibiting MAVS-mediated antiviral signaling and evading the host immune response.^71^ Notably, SFTSV utilizes NP and Gn, rather than NSs, to suppress the cGAS-STING signaling.^61^^,^^62^ SFTSV NP facilitates the interaction between cGAS and LC3, promoting the degradation of cGAS via autophagy.^61^ Furthermore, SFTSV Gn not only interacts with STING to degrade it through autophagy but also prevents its dimerization and inhibits K27-linked ubiquitination of STING, thereby disrupting the assembly of the STING-TBK1 complex.^62^ Mechanistically, regarding the induction of autophagy by SFTSV NP, it induces Beclin1-dependent autophagy by disrupting the Beclin1-BCL2 interaction.^54^ Additionally, SFTSV NP translocates to mitochondria by interacting with TUFM and mediates mitochondrial sequestration into phagophores through interaction with LC3, thus inducing mitophagy.^61^ Importantly, the N-terminal LC3-interacting region (LIR) motif of NP is crucial for the induction of mitophagy.^61^^,^^71^ Beyond its role in autophagy induction, SFTSV NP interacts with hnRNPA2B1, facilitating its retention in the cytoplasm, where hnRNP A2B1 binds to the 5′ untranslated region (UTR) of SFTSV RNA, thereby promoting viral RNA replication and SFTSV infection.^72^ Notably, this proviral mechanism exhibits evolutionary conservation across diverse RNA viruses, including VSV, SeV, EV71, and ZIKV.^72^

**Figure 3:**
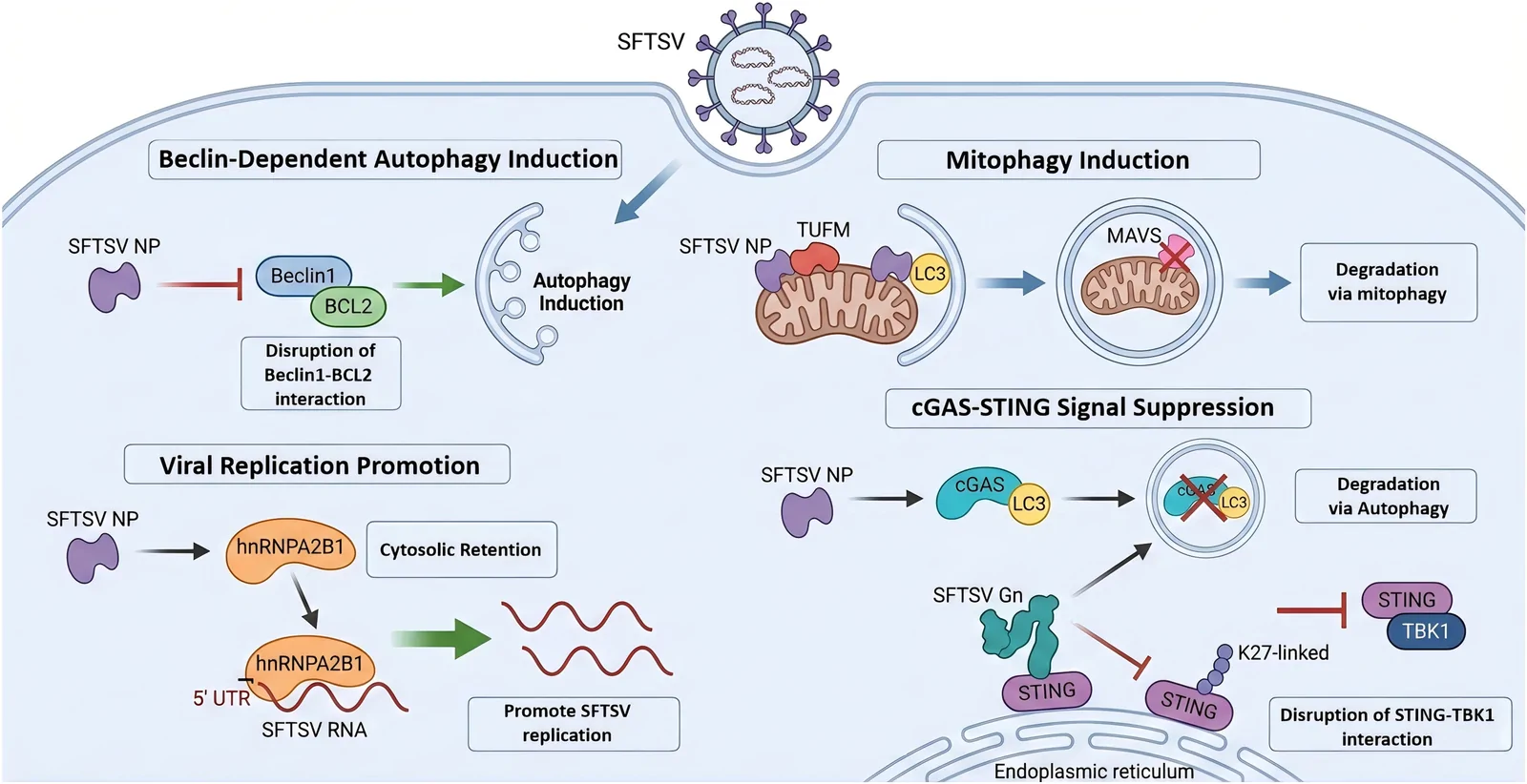
Immune evasion of SFTSV via Gn and NP SFTSV NP suppresses cGAS by facilitating its LC3-dependent autophagic degradation and inducing Beclin1-dependent autophagy/TUFM-dependent mitophagy. Simultaneously, Gn targets STING by promoting its degradation, preventing STING-TBK1 complex assembly, and inhibiting K27-linked ubiquitination.

## Role of SFTSV in modulating the inflammatory response

A key pathogenic mechanism contributing to the severity of SFTS is the development of a cytokine storm,^13^^,^^59^^,^^73^^,^^74^ while therapeutic suppression of inflammatory pathways through NF-κB inhibitors, p38 inhibitors, or NLRP3 silencing reduces viral replication.^36^^,^^75^^,^^76^ Recent studies indicate that SFTSV infection prompts the production of IL-1β predominantly via the activation of the NLRP1, NLRP3, and CARD8 inflammasomes.^35^^,^^36^^,^^59^^,^^77^^,^^78^ Mechanistically, oxidized mtDNA in the cytosol binds to and activates the NLRP3 inflammasome, though canonical NLRP3 triggers like calcium/potassium flux remain uninvolved^59^^,^^77^(Figure 4). Recent research provides different evidence concerning the mechanisms by which SFTSV NSs promotes inflammasome activation and associated inflammatory responses.^35^^,^^36^^,^^77^ The overexpression of NSs and other viral components, such as RdRp, Gn/Gc, and NP, fails to activate NLRP3.^77^ Further studies indicate that the N-terminal fragment of NSs interacts with NLRP3, facilitating the formation of the inflammasome complex in human microglial cells.^36^ As for NLRP1 and CARD8, SFTSV NSs disturbs the NLRP1/CARD8-DPP8/9 ternary complex and promotes the degradation of DPP8 and DPP9 for inflammasome activation in primary keratinocytes.^35^ Additionally, NSs can enhance NF-κB activation under SeV infection or TNF-α treatment by disrupting the interaction between TBK1 and IKKβ, thereby promoting NF-κB-mediated inflammatory responses.^79^ Unexpectedly, evidenced also showed that SFTSV NSs targets ABIN2, promoting the activation of the TPL2/ABIN2/p105 complex, which leads to the production of the anti-inflammatory cytokine IL-10, thus attenuating innate immune responses^37^(Figure 4), suggesting that SFTSV NSs may play a complex role in regulating the immune response to infection. Rather than being contradictory, this dual ability of NSs to simultaneously drive pro-inflammatory cascades and induce immunosuppressive IL-10 highlights a sophisticated viral strategy of immune dysregulation.

**Figure 4:**
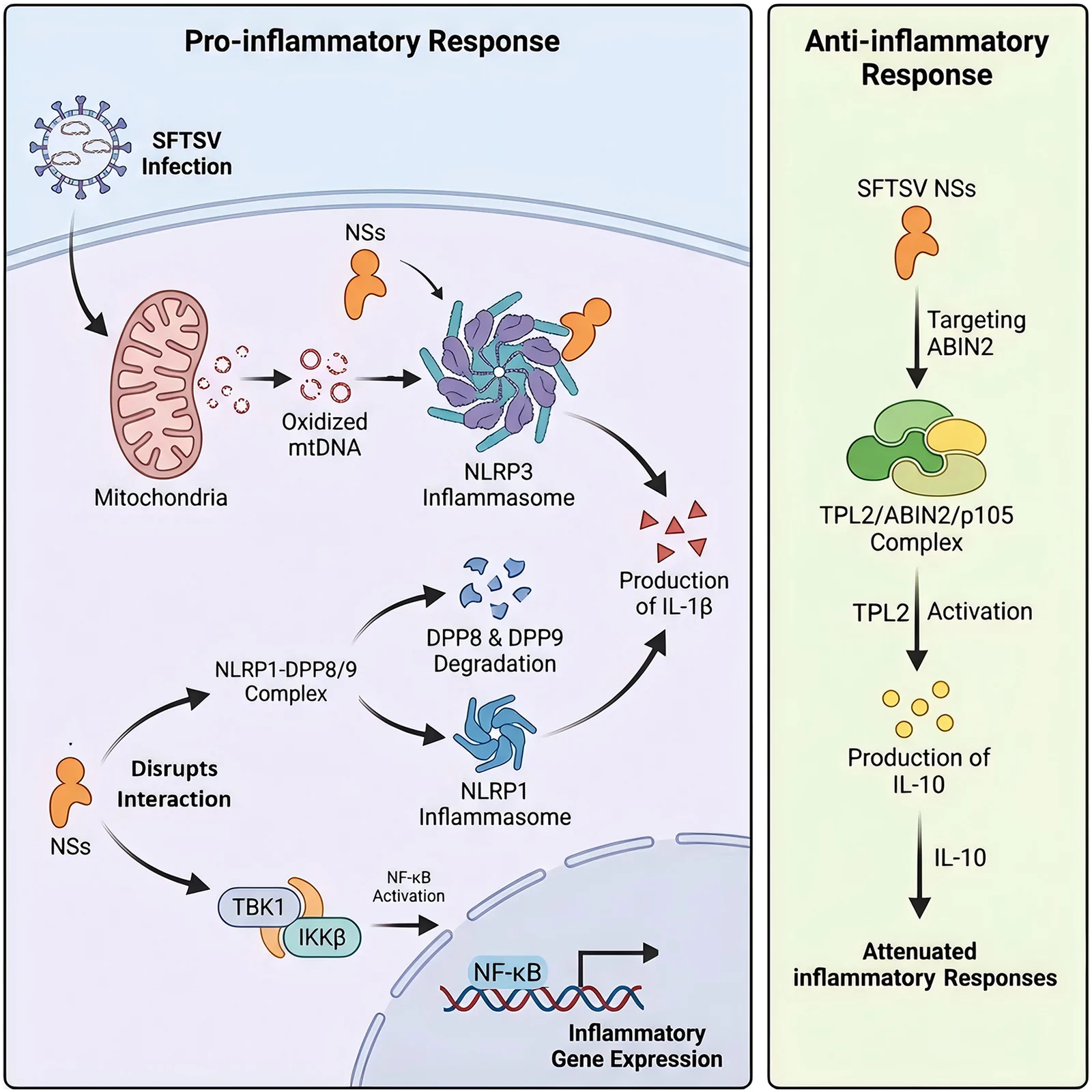
Dual role of SFTSV NSs in inflammation modulation SFTSV NSs plays a multifaceted role in the host inflammatory response. On one hand, it triggers pro-inflammatory pathways by activating the NLRP1, CARD8, and NLRP3 inflammasomes, or by enhancing NF-κB signaling. On the other hand, it facilitates an anti-inflammatory response by targeting TPL2/ABIN2/p105 complex, which subsequently promotes the production of the cytokine IL-10.

## Bile acid metabolism in SFTSV infection

Beyond classical intracellular immune sensors, systemic metabolic pathways, particularly the gut microbiota-bile acid axis, have recently emerged as critical modulators of the host innate immune response and SFTSV pathogenesis.^80^^,^^81^ Bile acid metabolism has been identified as a crucial regulator in the limitation of viral infections.^82^^,^^83^ Clinical studies indicate that taurolithocholic acid (TLCA) is associated with improved survival rates and decreased viremia in patients with SFTS.^80^ Mechanistically, TLCA exerts its antiviral effects through an innovative ferroptosis-dependent pathway, distinct from type I IFN signaling. The TGR5-PI3K/AKT-SREBP2 signaling axis promotes the expression of FADS2, thereby inducing ferroptosis, which effectively inhibits viral replication. This discovery is consistent with clinical observations that early increases in serum iron and ferritin, which correlate with reduced TLCA levels, are associated with fatal outcomes in SFTS patients.^80^ Notably, both ferroptosis inhibitors and TLCA have demonstrated protective efficacy in murine models of lethal SFTSV infection, underscoring the therapeutic significance of this pathway.^68^^,^^80^ Furthermore, *Akkermansia muciniphila* and its metabolite harmaline have been shown to enhance the production of chenodeoxycholic acid derivatives (GCDCA and TCDCA), which mitigate systemic inflammation through the inhibition of the TGR5-NF-κB pathway.^81^ The gut microbiota-bile acid axis further influences the pathogenesis of SFTSV through distinct mechanisms. Notably, an increased abundance of *Akkermansia*, *Lactobacillus*, *Enterococcus*, and *Parabacteroides* has been observed in patients with SFTS.^81^ Specifically, *A*. *muciniphila* was found to be more prevalent in surviving patients compared to those who succumbed to the disease.^81^ Additional studies have demonstrated a reduction in *Lachnospiraceae*, *Ruminococcaceae*, and *Sutterella* in SFTS patients, whereas opportunistic pathogens such as *Enterococcus*, *Streptococcus*, *Escherichia*, and lactate-producing bacteria like *Lactobacillaceae* were elevated.^84^

## SFTSV therapeutic strategies

SFTSV presents substantial challenges to public health prevention and control efforts. Existing treatment modalities are predominantly supportive, highlighting the critical need for innovative antiviral strategies. Focusing on the immune evasion mechanisms of SFTSV offers a promising avenue for therapeutic intervention. Current therapeutic strategies for SFTSV aim to disrupt various stages of the viral life cycle, primarily through both virus-directed and host-directed approaches. To block viral entry, targeting the LRP1 receptor, SFTSV entry factor, and utilizing neutralizing antibody cocktails has demonstrated encouraging therapeutic success in reducing lethality from SFTSV challenge, yet large-scale clinical trials remain absent.^46^^,^^85^^,^^86^^,^^87^^,^^88^ Meanwhile, efforts to inhibit viral replication using traditional antivirals have yielded limited clinical efficacy. Ribavirin, a traditional antiviral agent, exhibits limited efficacy, particularly in patients with elevated viral loads,^89^ while favipiravir, a broad-spectrum RNA polymerase inhibitor, has shown potential in reducing mortality when administered early; however, its benefits appear restricted to younger patients with low baseline viremia.^90^^,^^91^ Host-directed therapies that target immune pathways (e.g., arginine supplementation, calcium channel blockers) have shown promising results in inhibiting SFTSV infection.^92^^,^^93^ Given the central role of autophagy in both viral replication and immune evasion, targeting the autophagic flux using specific inhibitors could restrict viral assembly and egress.^54^ Moreover, the discovery of the microbiota-bile acid axis offers novel metabolic interventions. Supplementation with specific bile acids or probiotics could limit viral spread.^80^^,^^81^ Ultimately, while these diverse therapeutic avenues hold great promise, further research is essential to fully elucidate SFTS pathogenesis and translate these findings into effective clinical treatments.

## Adaptive immune responses during SFTSV infection

While innate immunity provides the first line of defense against SFTSV, coordinated adaptive immune responses are equally critical for viral clearance and disease outcome. The total number of T lymphocytes, especially CD4^+^ T cells, is markedly reduced in SFTS patients, with the extent of reduction positively correlating with disease severity.^94^ In addition, SFTSV targets plasmablasts, which become hyperactivated. Fatal cases display excessive plasmablast expansion yet fail to mount virus-specific IgG responses.^95^^,^^96^ Disrupted germinal center formation, impaired T follicular helper (Tfh) cell function, and a shift toward extrafollicular antibody production collectively contribute to low-affinity antibody generation and increased autoantibody formation, while cytokine storms further suppress both B and Tfh cell function. Mechanistically, this humoral collapse is driven upstream by SFTSV-induced monocyte apoptosis. Neutralizing antibody titers correlate with survival, and delayed or absent anti-Gn IgG responses are associated with poor prognosis.^95^^,^^96^ SFTSV further dismantles the adaptive immune landscape by depleting dendritic cells, polarizing macrophages toward the immunosuppressive M2 phenotype, and reducing arginine availability, thereby limiting T cell IFN-γ production and cytolytic capacity.^97^ Taken together, SFTSV orchestrates a multifaceted adaptive immune collapse, simultaneously impairing T cell activation, germinal center formation, and neutralizing antibody production, and reversing this immunosuppressive state remains a central challenge for immunotherapy development.

## Conclusion

This study delivers a comprehensive review of the current understanding of the sophisticated arms race between SFTSV and the host innate immune system. SFTSV employs a multipronged strategy to secure its survival, leveraging its NSs, NP, and Gn proteins to systematically dismantle host defenses. A central theme in SFTSV pathogenesis is the hijacking of autophagic pathways, which serves a dual purpose: facilitating viral assembly and egress while selectively degrading key antiviral sensors such as MAVS and cGAS. Despite these advances in understanding, several avenues require further exploration.

1 The host utilizes a variety of pattern recognition receptors to recognize SFTSV infection. One of the main challenges is understanding how SFTSV interacts with the host immune system for immune escape, particularly the innate immune system. SFTSV has developed multiple immune escape mechanisms, including blocking or hijacking cellular responses directly or indirectly for the viral life cycle during coevolution. Therefore, a comprehensive understanding of virus-host protein interactions is essential for the development of antiviral therapies.
2 Although research has largely focused on the role of NSs in immune evasion, emerging evidence indicates that NP and Gn also play significant roles in countering antiviral defenses. Nonetheless, these mechanisms remain incompletely understood and warrant further investigation.
3 SFTSV infection triggers multiple classical cell death pathways. Although autophagy is well characterized, the interplay between SFTSV and other programmed cell death pathways remains unexplored. The revelation that TLCA-induced ferroptosis restricts viral replication suggests that metabolic regulation of cell death could be a potent therapeutic target. Furthermore, the role of autophagy in SFTSV-induced immune responses is not fully understood. While autophagy has been shown to play a key role in bunyavirus assembly and release, the specific mechanisms involved in these processes are not well characterized.
4 The emerging role of the gut microbiota-bile acid axis offers a novel frontier for intervention. It is worth noting that the intestine is recognized as the primary target organ of SFTSV. Future research should prioritize dissecting the specific metabolites and microbial communities that confer protection, potentially paving the way for microbiota-based therapeutics or metabolite supplementation as adjunctive treatments for SFTS.

## Acknowledgments

This study was supported by the National Natural Science Funds of China (82302523) and the Natural Science Foundation of Hebei (H2023206290); 10.13039/501100003819Hubei Provincial Natural Science Foundation (2023AFB910); 10.13039/100016083Hebei Provincial Department of Human Resources and Social Security (A20240020); Hebei Municipal Government Funded the Training of Clinical Medical Talents (ZF2025063); Key Science and Technology Support Program Project of Hebei Province (242W7702Z); the Natural Science Foundation of Hebei (H2025206425); Key Research and Development Project of Universities in Hebei Province (241460083A); Public Welfare Empowerment Special Clinical Research Project (BHCF-EH-2025-8, BHCF-EH-2025-9). The funders had no role in the study design, data collection, and analysis, decision to publish, or the preparation of the manuscript.

## Author contributions

X.M., Y.S., and Z.-M.W.: writing – original draft and supervision; X.J. and C.-m.Z.: supervision; B.P., L.K., and L.S.: writing – review and editing; All authors read and approved the final manuscript.

## Declaration of interests

The authors declared that they have no conflict of interest.
